# The influence of recent bushfires on water quality and the operation of water purification systems in regional NSW

**DOI:** 10.1038/s41598-024-66884-3

**Published:** 2024-07-13

**Authors:** Reed Jackson, K. C. Bal Krishna, Miao Li, Arumugam Sathasivan, Lalantha Senevirathna

**Affiliations:** 1 School of Computing, Mathematics and Engineering, Charles Sturt University, Bathurst, NSW Australia; 2 School of Engineering Design and Built Environment, Western Sydney University, Penrith, NSW Australia; 3 Gulbali Institute for Agriculture, Water and Environment, Charles Sturt University, Albury, NSW Australia

**Keywords:** Bushfire, Water quality, Temperature, Water treatment, Regional Australia, Environmental chemistry, Environmental impact, Chemical engineering, Civil engineering

## Abstract

Over the past decade, escalating extreme weather events have significantly affected New South Wales (NSW), Australia, with unprecedented droughts and intense fires. Yet, the impact on water quality and purification processes remains insufficiently studied. This research focuses on the immediate changes in NSW's environmental water quality and issues in water purification unit operations following the 2019 bushfires. Water samples and maintenance records from affected catchments, intakes, purification units, and reservoirs were analysed. Compared to control samples, post-bushfire water exhibited high turbidity. Sediment and ash shock loads posed significant threats to aquatic ecosystems. Elevated turbidity, suspended sediments, pH, and alkalinity were major concerns for water purification. Raw water samples showed turbidity exceeding 195 NTU, with flocculation and sedimentation most impacted. Immediate measures included sediment traps, aeration, pre-chlorination, and inline monitoring. These findings inform strategies to mitigate bushfire impacts on water quality and optimise water purification in fire-prone regions.

## Introduction

Forest catchments are a vital source of raw water for town water purification systems due to their superior water quality parameters^1^. About one-third of the world’s largest cities and numerous regional towns rely on protected and dedicated forest catchments for a significant portion of Ftheir drinking water^2^. However, the increasing frequency of large bushfires in recent decades has highlighted the challenges in ensuring a safe and uninterrupted water supply for residents of both regional and big cities^3–7^. For instance, from 2003 to 2007, over 3 million hectares of forest in southeast Australia were destroyed by bushfires^8^. Extensive areas along the eastern seaboard of Australia were engulfed in flames during the spring and summer of 2019–2020, resulting in a devastating impact. The New South Wales (NSW) Rural Fire Service bore the brunt of this catastrophe, with approximately 7% (5.37 million hectares) of land in NSW being severely affected. The fire ground encompassed 37% of the national park estate, including all categories of parks. Also, 42% of state forests and 4% of freehold land were within the fire ground’s reach. A closer examination of the land within the affected area reveals that national parks accounted for 50%, state forests comprised 17%, and freehold land constituted 29% of the overall composition. These figures highlight the widespread impact of the fires, with significant implications for the environment and communities^9^. Moreover, the destruction of the fire ground had adverse effects on the drinking water supplies of major cities, such as Melbourne and Sydney, as well as numerous regional towns.

Numerous studies have reported abrupt changes in catchment water quality following major bushfires. These changes include elevated levels of sediments, nutrients, and organic pollutants that diminish primary and secondary water quality parameters, potentially creating unfavourable conditions for traditional water purification processes^10–14^. For example, heavy thunderstorms followed the bushfires in late February and March 2020, resulting in the erosion of ash, fire debris, and higher sediment from the bushfire-affected reservoirs and river catchments^15,16^. This not only disrupted the water supply but also posed long-term systemic risks to drinking water production^17–19^. While limited available literature has suggested modifications to treatment unit operations and operational procedures due to the impact of extreme weather events, including bushfires, on municipal water treatment^7,20–22^, further research is needed to explore the impact of post-bushfire runoff on water bodies^5,6,23^ and the specific challenges faced by water treatment plant operators after major bushfires.

This study aims to investigate: (a) the impact of the 2019–2020 bushfires on water quality in the mid-western region of NSW, Australia, (b) the effect of catchment water quality on the water treatment plant’s performance, and (c) the challenges faced in the water treatment plant operation. In this study, water samples were collected from several locations in the catchment area and analysed for various water quality parameters, including pH, temperature, turbidity, colour, alkalinity, and hardness. Further, challenges faced by the treatment plant operator producing the water complying with drinking water quality as a result of heavy rainfall after the bushfires on catchment water quality. This study emphasises the importance of understanding the impact of bushfires on water quality and the need for appropriate management strategies to mitigate the risks. The findings can benefit not only the regional NSW but also other regions vulnerable to bushfires globally.

## Methodology

### The study area, the plant and sampling

The selected water treatment plant supplies drinking water to a population of 30,000 in a regional town located about 280 km northwest of Sydney. The area is predominantly rural and covered by heavy timber through to open grassland. The landform ranges from 350 to 1070 m elevation. There are about 1145 km^2^ of forest and national parks with catchment areas of 8700 km^2^. The main local catchment area is associated with the Cudgegong River system, and forms on the western side of the Great Dividing Range. The varied landforms and altitudes over the area indicate that there are localised variations to the climate patterns. Temperature can vary from as low as − 12 °C in winter to high 30 s or low 40 s °C in summer. The hottest months are November to February, and the coldest months are June and July. In higher altitude areas, snowfalls can occur in winter. Rainfall ranges from 68 mm in January to 44 mm in April, with an average rainfall for the area being 668 mm.

The raw water for this plant is sourced from a dedicated reservoir, which features a concrete arch dam flanked by earthfill embankments on either side, holding approximately 3000 ML of water. The reservoir is ungated and the reservoir overflow is uncontrolled. The catchment area encompasses a variety of land uses, including olive oil production, farms for sheep, cows, and pork, vegetable farms, vineyards, and a fig orchard. Over the past 70 years, significant floods occurred in 1955, 1969, 1971, 1974, and 1990. Additionally, major floods occurred in February 2003 and December 2010 within the same catchment. More recently, in 2016 and 2017, the area experienced smaller-scale floods.

The catchment area suffered severe impacts from the 2019–2020 bushfires, with more than 70% of it being burnt by the flames. From January 2020, several fires started in the catchment but were brought under control. The main fire started in late January and lasted until 7 February; it impacted over 150,000 hectares. High rainfall after the fires produced a large pollution load, which drastically changed the raw water quality parameters, leading to water purification challenges. During the severe drought period, the reservoir water level plummeted to 30%. However, following the cessation of the drought and the onset of heavy rain, the level surged to 55% within 7 days.

To ensure the quality of the water supply, the reservoir is strictly protected. Human activities like swimming, vehicles, firearms, stock and domestic animals, and watercraft are prohibited. The water purification process includes initial filtration (fine mesh screening), pH adjustment, coagulation, flocculation, filtration (sand and crushed coal), neutralisation (adjusting the pH for distribution), chlorination (disinfection), and fluoridation (to prevent dental decay). Water samples were collected from various points, including the reservoir (30 samples after the bushfires and 24 samples before the bushfires), treatment unit processes (up to 40 samples), and clean water reservoirs (up to 90 samples). This sampling started on 20 January 2020 and continued until 15 March 2020. Moreover, available raw water and treated water quality data extracted from the local council database for one year (April 2019 to April 2020) were analysed in this study.

### Analytical methods

The standard titration method (method 2320) was used to determine both the alkalinity (hydroxide, bicarbonate, carbonate) and total alkalinity as CaCO_3_, while the ion chromatography method was used to measure sulphate and chloride levels. The fluoride level was determined with an ion-selective electrode. To measure other parameters such as pH, temperature and conductivity, levels, a portable multiparameter meter (Instrument Choice™ HI98194) was used. The colour and turbidity of the water were measured using a HACH DR1900 and a HACH 2100Q turbidity meter, respectively. The collected samples were analysed within 24 h post-collection and were tested in batches of ten to ensure accuracy. Duplicate samples and matrix spike recoveries were within the laboratory acceptance criteria. Finally, the water treatment plant logbook and daily maintenance records were consulted to identify specific challenges encountered and technical and operational protocols followed during extreme weather events.

### Statistical analysis

The data were analysed using IBM SPSS Statistics (version 27, IBM SPSS Inc., Chicago, IL). The normality of the data was assessed using the Shapiro-Wilks test, as well as a visual inspection. The means and standard deviations, or the median and interquartile range, were used to present the data distribution pattern. The minimum and maximum values were also provided for some of the water quality data. To determine if there were significant differences in water quality parameters before and after the bushfires, an independent sample t-test was used for statistical comparison. The significance level was set at ≤ 0.05. When calculating the mean values, target analytes with concentrations lower than the LOD (limit of detection) were considered to be one-half of the detection limit. This approach was adopted to ensure that the target analytes with low concentrations were accurately represented in the analysis.

### Consent to publish

All authors agreed with the content, gave consent to submit, and obtained consent to publish from their university.

## Results and discussion

### Impacts on water quality

Raw water quality parameters (pH, turbidity, alkalinity, colour, and hardness) were monitored before and after the bushfire and rainfall events (Table 1). The bushfire and rainfall events impacted water quality, especially turbidity, pH, alkalinity, and colour (Table 1).

**Table 1:** Changes in raw water quality parameters before and after the bushfire and rainfall events.

		pH	Turbidity (NTU)	Colour (Pt-Co)	Alkalinity (mg-CaCO_3_/L)	Total hardness (mg-CaCO_3_/L)
After bushfire and rainfall	Mean	7.78	32.34	220.55	159.73	216.27
	N	30	30	22	15	15
	SD	0.25	37.11	91.07	13.83	17.28
Before bushfire and rainfall	Mean	7.46	18.57	263.61	146.55	219.45
	N	24	24	18	11	11
	SD	0.17	8.95	95.89	7.90	16.57
Total samples (before and after bushfire)	Mean	7.64	26.22	239.93	154.15	217.62
	N	54	54	40	26	26
	SD	0.27	28.91	94.58	13.28	16.72

*N* The number of water samples, *SD* Standard derivation.

**Figure 1:**
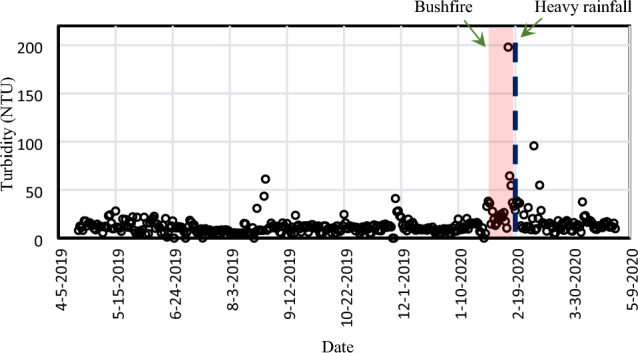
Changes in raw water turbidity before and after the bushfire and rainfall events.

Figure 1 visually presents the temporal variation of raw water turbidity. To encompass the span of 12 months, the plot integrates water quality data from the council’s database. Notably, as depicted in Fig. 1, the maximum turbidity reached 195 NTU following a significant bushfire in January 2020, representing a level 15 times higher than the average. Most turbidity levels reported in February and March 2020 were also above average. Before the bushfire and rainfall events, the most frequent turbidity level observed in the study period was 10 NTU, with a mean of 18.57 NTU and SD of 8.95 NTU. The turbidity level took about four weeks to reduce from 195 to 12 NTU. Substantial peaks in turbidity and nutrients after major bushfires have been reported in past studies. For example, turbidity levels of over 1000 NTU were reported after a bushfire in the Murray-Darling Basin^24,25^, which is much higher than would be expected for such systems. Based on the frequency of turbidity raw water levels measured during 2019–2020, the probability of exceeding the raw water turbidity level was < 5%, and most of the time, the turbidity level fluctuated around 12 NTU, as shown in supporting information Fig. SI-1.

Turbidity indicates the concentration of suspended solids in water, such as clay, organic matter, and inorganic matter, which often correlates with other pollutants in water^26^. The ash and debris from the burnt vegetation can be carried into streams and waterways by runoff, which increases the concentration of suspended solids in water^27^. In addition, the high temperature of the bushfires can cause the organic matter in the soil to be released into the water, which can also increase turbidity levels^28^. Elevated turbidity levels can have several negative effects on water quality, such as reducing light penetration, altering aquatic habitats, and affecting the ability of aquatic organisms to feed and respire.

Many studies have reported the transportation and deposition of organic and inorganic sediments from bushfire-affected catchments, causing immediate major water quality problems, particularly unmanageable turbidity peaks^29^ faced by water treatment plant operators. Fluctuations in raw water turbidity significantly impact the operating performance of drinking water treatment plants as various process parameters must be adjusted, for example, the dosage rate of coagulants and polymers, operating flow rates, and filter backwash frequencies. In municipal water supplies, turbidity is considered a surrogate measurement for both microbiological contamination and water clarity^30^.

**Figure 2:**
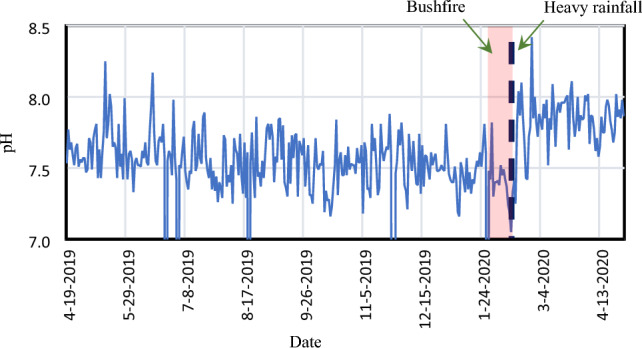
Change in raw water pH before and after the bushfire and rainfall events.

Figure 2 demonstrates the impact of bushfires on the pH of raw water. The Figure incorporates water quality data from the council’s database to depict the annual variation. Before the major bushfire in 2019, pH levels were fluctuating around 7.5. The raw water pH level suddenly increased from 7.1 to 8.4 within a few days after the bushfire and then fluctuated around 7.8 for the rest of the year, which is similar to past bushfires in Australia^31^. One possible reason for this spike is humic acid, which leaches from bushfire residues^32^. The solubility of ash and charcoal in water is proportional to the pH level, which ultimately affects the raw water pH level^33^. Supporting this study, Law and Evens^34^ reported the leaching of metal hydroxide and oxides, including calcium and manganese compounds, and increased water pH. Further, Yusiharni and Gilkes^35^ also reported the soil pH level changed from 7.0 to 9.0 after a major bushfire at Wundowie, Darling Range, Western Australia. The increase in water pH depends on the catchment area, bushfire intensity, bushfire area, rainfall intensity, and duration, etc.^31^. Further, the changes in raw water pH affect the unit treatment processes, such as coagulant and flocculant dosages, disinfection efficiencies, and disinfectant stability.

An increase in alkalinity was noted as the result of bushfire and rainfall events (Table 1). Water alkalinity levels increase after a major bushfire due to dissolved ash and other burned organic matter in the water. The ash and burned vegetation that enter waterways following a bushfire can contain significant amounts of calcium, magnesium, and other metals, which can react with the water to increase alkalinity levels^36^. Moreover, the increased concentration of alkaline minerals in the water can act as a buffer and prevent the water from becoming too acidic. Demeyer et al.^37^ reported that CaCO_3_ is the major compound of ash, along with other carbonates and oxides of metals and silica. Wood ash is highly alkaline, increasing soil pH by up to three units immediately after burning compared to unburned soils^38^, which could be the reason for the increased alkalinity after the bushfire.

After the bushfire and rainfall events, low average colour was noted (Table 1). The colour in water is due to the dissolved compounds and the reasons for the lower colour after the bushfire and rainfall events are unknown. The raw water temperature experienced a sudden increase of 3 °C (from 24 to 27 °C) with the fresh rainfall after the bushfire. However, over the following 12 weeks, it gradually decreased. A past study^39^ also reported a maximum temperature of 17.2 °C, which was 7.8 °C above the maximum recorded in a nearby unburnt stream based on bi-hourly measured water temperatures during a bushfire in Montana, USA. Further, during the Black Saturday bushfires in Victoria (February 2009), water temperatures in streams and ponds were reported to have reached 55 °C^40^. The difference in sampling and the testing period could be the reasons for not noticing the difference in water temperature before and after the bushfire and rainfall events in this study. Despite the bushfire and rainfall events, no significant changes were observed in the alkalinity and total hardness of the raw water. Figure SI-2 represents the temporal variations of these parameters.

### Correlation between water quality parameters

By using statistical comparison (Spearman’s correlation) of raw water quality parameters, the water temperature was identified as the most significant parameter. The temperature showed a statistically significant (*p* < 0.01) correlation with pH, colour, calcium hardness, and turbidity (Table 2). The temperature was positively correlated with colour and turbidity but negatively correlated with pH, alkalinity, and calcium hardness. A higher temperature shifts the reaction HCO_3_^−^⇔CO_3_^2−^ + H^+^ to the right, which increases the acid (H^+^) concentration and causes a slight drop in pH, and hence a significant negative correlation between pH and temperature. The water chemistry strongly correlates with water temperature and the rate of chemical reaction generally increases with the temperature^41^. Further, water with high temperatures dissolves more minerals from the surrounding rocks and will, therefore, have a higher colour and conductivity, resulting in a significant positive correlation of temperature with colour and turbidity. Temperature is an important environmental water quality parameter as it relates to many important processes and quality parameters, such as biological processes, water density, stratification, and dissolved oxygen level^42,43^.

**Table 2:** Correlation between water quality parameters in this study.

		Temperature	pH	Colour	Total hardness	Calcium hardness	Alkalinity
pH	Correlation coefficient	− .189					
	Sig. (2-tailed)	< 0.01					
	Number of samples	616	617				
Colour	Correlation coefficient	.309	− .063				
	Sig. (2-tailed)	< 0.01	.156				
	Number of samples	504	505	505			
Total hardness	Correlation coefficient	− .073	− .146	.044			
	Sig. (2-tailed)	.149	.004	.393			
	Number of samples	389	389	386	389		
Calcium hardness	Correlation coefficient	− .219	.082	− .090	.403		
	Sig. (2-tailed)	< 0.01	.106	.076	< 0.01		
	Number of samples	388	388	386	388	388	
Alkalinity	Correlation coefficient	− .045	.241	− .256	.041	.051	
	Sig. (2-tailed)	.379	.000	.000	.425	.320	
	Number of samples	384	384	382	384	384	
Turbidity	Correlation coefficient	.220	.001	.691	.020	.018	− .259
	Sig. (2-tailed)	< 0.01	.985	< 0.01	.699	.718	< 0.01
	Number of samples	616	617	505	389	388	384

The alkalinity of water is its ability to neutralise acids, which is why there is a significant positive correlation between pH and alkalinity. In the water treatment process, a minimum alkalinity of > 60 mg-CaCO_3_/L maintains water pH within the water distribution networks. Colour has a significant negative correlation with alkalinity and a positive correlation with turbidity. Colour in the water results from various sources, including natural metallic ions, plankton, and weeds, a byproduct of the degradation of plants and organisms, which results in a positive correlation between colour and turbidity. The total hardness of the water is the measure of the cations with a charge of two or more multivalent positively charged cations (such as calcium, magnesium, etc.), which results in a significant positive correlation between calcium hardness and total hardness (Table 2).

### Challenges in the treatment process and strategies to overcome these issues

**Figure 3:**
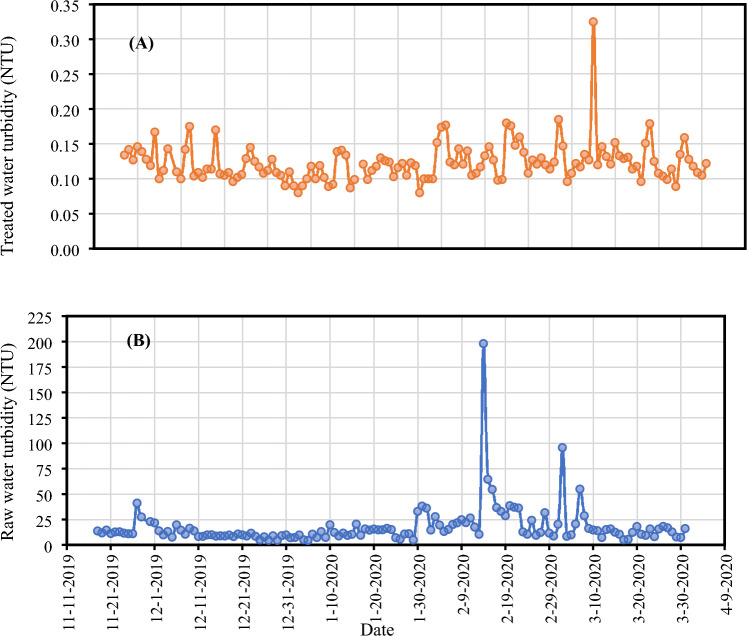
Turbidity levels in treated (**A**) and raw (**B**) water.

Despite the challenges in treating source water with extreme quality parameters during and after the major bushfire, treated water turbidity was < 0.4 NTU (Fig. 3) during this study period. However, there were several challenges to achieving this level of turbidity. When the turbidity was high due to bushfires and storms, 3000 m^3^/day of groundwater, about 25% of the total demand, was mixed with surface water. The groundwater has higher hardness than the surface water (Fig. SI-3).

After mixing groundwater (25% of the total demand) with surface water, the turbidity peak in raw water affected the filtration plant’s ability to adequately treat the water according to the Australian Drinking Water Guidelines (ADWG, SI-4). Experienced engineers and operators had to conduct several jar tests with highly turbid water to adjust the coagulant and polymer dose and could not achieve any floc formation. There was no flocculation, and the filtered water turbidity was high and brown on settling.

After two days of extensive efforts to meet the water quality standards outlined in the ADWG, plant managers observed that switching from Alchlor Gold to aluminium sulphate as a flocculant produced improved results. Regrettably, this change couldn’t be implemented at the time due to logistical challenges, including timely delivery of alum sulphate, storage constraints, and the availability of suitable pumping facilities. This experience underscores the critical need for process flexibility and streamlined operational procedures. For instance, plant operators should be adequately trained to select the most appropriate flocculant based on the specific conditions, and essential chemicals should be readily accessible in the inventory to ensure a timely response to such operational requirements.

After several tests and consultations, the raw water alkalinity was identified as the main reason for not meeting the required flocculation efficiency. Although groundwater constituted 25% of the total water, the high alkalinity level in the groundwater chemically unbalanced the flocculator, minimising the formation of healthy flocs. Further published studies have shown the influence of total hardness on the coagulation process and the increase in reaction time with the total hardness level^44^, which could also be another cause for the lack of a good floc formation.

The high alkalinity of the groundwater meant all unit operations were stopped and the entire treatment process was restarted with only surface water. A jar test determined an effective coagulant dose to achieve turbidity of < 0.2 NTU in filtered water. The critical control limit of filter water turbidity is < 0.2 NTU to prevent the risk of escaping *Cryptosporidium* and *Giardia* through the filter. The plant was shut down four hours later for a restart the next morning. The reason for this was to monitor any changes and minimise fatigue risk. However, some of the water from the clear water tank had been pumped into town, and the council issued a boiled water alert following consultations with the NSW EPA (Environment Protection Authority) and NSW DPIE (Department of Planning and Environment). NSW EPA boiled water alerts are issued when there is the detection of *E*. *coli* and poor raw water quality or when treatment or disinfection critical limits are exceeded. In this case, a boiled water alert was issued due to the poor water quality despite free chlorine residuals being 2.0 mg/L in the clear water tank. The free chlorine residual profile is presented in Fig. SI-4.

The council undertook reticulation testing, which indicated a turbidity level varying from 0.02 to 1.0 NTU. This was the only failure in terms of meeting the ADWG. However, boiled water alters were noticed and there were no *E. coli*. The testing was carried out for ten days; the turbidity was reduced, and chlorination levels were adequate in the reticulation.

### Treated water quality parameters

The quality of treated water is highly influenced by source water quality parameters^45^. Descriptive statistics of treated water quality during the 2019–2020 summer are shown in Table 3. Irrespective of the poor source water quality observed after the bushfire, utility operators managed to maintain the treated water quality to the required standards.

**Table 3:** Treated water quality parameters.

	N	Minimum	Maximum	Mean (SD)	Skewness (SE)	Mode	Percentiles		
							25	50 (Median)	75
Temperature (^o^C)	89	19.1	28.7	22.7 (1.7)	0.6 (0.3)	21.6	21.6	22.6	23.7
pH	90	7.0	8.4	7.5 (0.3)	1.3 (0.3)	7.5	7.3	7.5	7.6
Chlorine (mg/L)	84	1.40	2.85	2.3 (0.3)	− 0.8 (0.3)	2.6	2.2	2.4	2.6
Colour (Pt-Co)	75	0.0	23	4.9 (3.5)	2.2 (0.3)	4.0	3.0	4.0	7.0
Total hardness (mg-CaCO3/L)	48	110	152	129.1 (11.0)	0.6 (0.3)	120^a^	120.0	127.0	136.0
Calcium hardness (mg-CaCO3/L)	48	66	180	89.7 (21.4)	3 (0.3)	88^a^	78.0	88.0	92.0
Alkalinity (mg-CaCO3/L)	48	28	100	52.9 (11.8)	1.6 (0.3)	46^a^	46.0	51.0	57.5
Turbidity (NTU)	90	0.0	1	0.1 (0.07)	7.9 (0.3)	0^a^	0.1	0.1	0.1
Fluoride (mg/L)	90	0.16	1.08	0.9 (0.2)	− 4.4 (0.3)	0.98^a^	0.9	1.0	1.0

^a^Multiple modes exist. The smallest value is shown.

The treated water turbidity ranged from < 0.1 to 1 NTU with a mean of 0.1 NTU. The 25, 50, and 75 percentiles of treated water turbidity showed 0.1 NTU (always less than 5 NTU as per the ADWG), suggesting this level was well maintained throughout the summer, except on a few occasions. Despite a higher fluctuation of source water pH around 7.9 in the summer, the fluctuation of pH in the treated water was minimal, with the mean (SD) 7.5 (0.3) which was within the ADWG range of 6.5–8.5. In the treated water, the mean colour is 4.9 Pt-Co, which is an important aesthetic characteristic for customer acceptance. Like turbidity, treatment processes are optimised to remove colour. Calcium hardness was between 66 and 180 mg-CaCO_3_/L, within the range of 60–200 mg/L as per the ADWG. Soft water (< 60-CaCO_3_ mg/L) may lead to greater corrosion of pipes, although this will depend on other factors such as pH, alkalinity, and dissolved oxygen concentration. Total hardness above 200 mg-CaCO_3_/L may lead to excessive scaling of pipes and fittings and cause the blockage of safety relief valves in hot water systems. Similarly, alkalinity was also in the range of 28 to 100 mg-CaCO_3_/L, with a mean of 52.9 mg-CaCO_3_/L.

**Figure 4:**
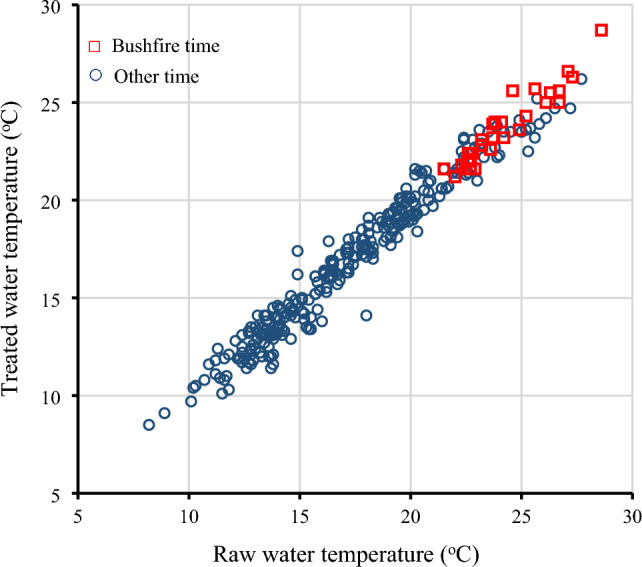
The relationship between treated water temperature and raw water temperature, emphasising the influence of the bushfire on water temperature.

Additionally, the raw water temperature showed a strong linear correlation with the treated water temperature (Fig. 4), suggesting potential impacts on water quality management and the integrity of water infrastructure. The impact of treated water temperature on public health and the performance of the water distribution system is poorly studied. However, most countries have maintained a drinking water temperature below 25 °C^46^. This study noticed that the treated water temperature exceeded 25 °C for 10 days during the major bushfire in 2020 (Fig. 4). The water temperature impacts disinfection stability and efficiency, including bacterial and biofilm regrowth.

### Limitations

A major limitation of the current study is the restricted number of water quality parameters that have been included in the analysis. Several vital parameters, such as total organic carbon (TOC), nitrogen (N), phosphorus (P), algal cells, iron, and manganese, have been omitted from the study. Previous research suggests that these parameters can be significantly impacted following a bushfire. Of particular importance is TOC, which plays a critical role in the design and functioning of water treatment systems. TOC measures the total amount of organic carbon present in water and is an essential parameter for evaluating the effectiveness of chemical dosing in water treatment systems. Its concentration in water can affect the efficiency of various water treatment processes, such as coagulation, flocculation, and disinfection, including the disinfection byproducts. Iron and manganese are two other critical parameters that are reported to increase in source water following bushfire and rainfall events^31^. Elevated concentrations of iron and manganese in drinking water degrade water quality by impacting the taste, odour, and colour^47^. Increased concentrations of manganese in drinking water have been associated with adverse health impacts, including neurological disorders^48^. The ADWG has established a health guideline limit of 0.5 mg/L manganese and an aesthetic limit of 0.3 mg/L iron. To remove and/or reduce the iron and manganese in water treatment plant, implementation of additional treatment processes such as aeration, chlorination and addition of potassium permanganate should be considered. Thus, the increase of iron and manganese in source water plays a significant role in the operation of the water treatment plant, including the upgrade and design.

Increased concentrations of limiting nutrients (nitrogen and phosphorous) in the dams and lakes after the bushfire cause the algal/cyanobacterial growth to persist for longer than usual^17^. High cyanobacterial biomass potentially affects the taste and odour of treated drinking water. Moreover, there is a great risk of releasing cyanobacterial toxins (*Cylindrospermopsin* and neurotoxins) in water distribution systems^49^. Algal blooms present challenges in water treatment processes, such as increased coagulant demand and membrane fouling and contributing to disinfection byproducts^50^. To reduce the effects of iron, manganese, and algae in water treatment plants, the plant operators usually change the water offtake level of raw water in the dam/reservoir, depending on the availability of the facility in the pumping stations.

Future studies should address these limitations by including a more comprehensive range of water quality parameters to improve the understanding of the impacts of bushfires on water purification.

### Preparedness to respond to the next extreme weather event

This study and past case studies have reported the direct impact of rainfall preceded by bushfires on source water and drinking water supplies. Impacted source water quality includes both secondary parameters (taste and odour) and primary parameters, such as increased turbidity and organic matter, and a change in pH, conductivity, and alkalinity. Therefore, to minimise the impact of the next extreme weather event on the town water supply, technical, operational, and management strategies were identified as listed below.

1. Rehabilitate paddocks adjoining the river as the drought denuded the vegetation and the bushfire prevented the natural vegetation from acting as a pre-filter to the river.
2. Council to investigate alternative water supply options and rectify a bore water supply to increase the available water yields and develop a treatment optimisation procedure considering high alkalinity.
3. Enhance the council's water quality monitoring and management strategy by integrating regular assessments of key parameters such as dissolved oxygen, metals, and nutrients in the source water.
4. The installation of inline monitoring devices for turbidity and pH, and facilities such as an aerator, KMnO_4_ dosing, and pre-chlorination to oxidise the iron and manganese.
5. Modify the treatment process by introducing an aerator and a powder-activated carbon dosing unit for algae and odour control.
6. Implement regular programs to train staff for better plant management and decision-making during water quality challenges, including training for optimising treatment processes (such as optimising the coagulant dose, adjusting pH, and using appropriate coagulant during challenging times).
7. Establish a mechanism for internal and stakeholder communications during emergencies.
8. Initiate research projects to understand the behaviour of the catchment, post bushfires erosion and debris flows and their impact on water quality and treatability and use the findings to develop a reliable risk management strategy.
9. To treat the highly turbid water, coagulant and flocculants concentrations are required to increase^45^. While increasing the chemical concretions, the residuals chemicals in the treated water should be monitored as outline in the ADWG. For examples, if alum is dosed as a coagulant, aluminium concentrations should be regularly monitored to keep its concentrations below 0.2 mg/L as there is a tentative link between the aluminium concentrations and Alzheimer’s disease^51^.

## Conclusions

This study aimed to investigate the impact of bushfire and rainfall events on the quality of raw water, as well as the correlation between various raw water quality parameters and the subsequent treatment process. Through a thorough analysis of the data, several conclusions were drawn.

Firstly, it was found that bushfire and rainfall events significantly impact the turbidity, pH, alkalinity, and colour of the raw water. The data showed that these events caused considerable changes in the water quality, which could negatively affect the treatment process if not managed properly. Secondly, it was observed that water temperature had a statistically significant correlation with pH, colour, calcium hardness, and turbidity. The data showed that temperature positively correlated with colour and turbidity but negatively correlated with pH, alkalinity, and calcium hardness. This information is critical for water treatment facilities to consider when designing and implementing their treatment processes. Additionally, the study found that good floc did not form when groundwater, which had higher hardness, was mixed with surface water. This information is crucial for water treatment facilities to consider when designing their treatment processes.

Despite the significant changes in raw water quality due to bushfire and rainfall events, it was found that the treated water quality met the ADWG. This indicates that the water treatment process effectively produced safe and clean drinking water for the town’s residents. Furthermore, it was observed that the raw water temperature showed a strong linear correlation with treated water temperature. This information can be used by water treatment facilities to adjust their treatment processes accordingly to maintain optimal treated water temperature. Finally, the study provided technical, operational, and management strategies to minimise the impact of the next extreme weather event on the town water supply. These strategies are crucial for water treatment facilities to ensure they can continue to provide safe and clean drinking water to the community, even during extreme weather events.

## Supplementary Information

Supplementary Information.

## Data Availability

The datasets used and analysed during the current study are available from the corresponding author on reasonable request.
