# Author Correction: The influence of recent bushfires on water quality and the operation of water purification systems in regional NSW

**DOI:** 10.1038/s41598-024-72927-6

**Published:** 2024-09-18

**Authors:** Reed Jackson, K. C. Bal Krishna, Miao Li, Arumugam Sathasivan, Lalantha Senevirathna

**Affiliations:** 1 School of Computing, Mathematics and Engineering, Charles Sturt University, Bathurst, NSW Australia; 2 School of Engineering Design and Built Environment, Western Sydney University, Penrith, NSW Australia; 3 Gulbali Institute for Agriculture, Water and Environment, Charles Sturt University, Albury, NSW Australia

Correction to: *Scientific Reports *10.1038/s41598-024-66884-3, published online 13 July 2024

The original version of this Article contained errors.

The name of author Arumugam Sathasivan was incorrectly given as Sathaa Sathasivan.

In addition, the Acknowledgements section was incomplete.

“The authors would like to thank Mr. Mark Filmer for his assistance in enhancing the readability of this manuscript.”

now reads:

“The authors would like to thank Mr. Mark Filmer for his assistance in enhancing the readability of this manuscript. This publication was supported by the CSU Open Access Publishing Scheme and the Next Generation Water Hub Project.”

The original Article has been corrected.

