# Supplementary material for: The influence of recent bushfires on water quality and the operation of water purification systems in regional NSW
Source: Sci Rep. 2024 Jul 13;14:16222. doi: 10.1038/s41598-024-66884-3 (PMC11246477; doi:10.1038/s41598-024-66884-3)
Supplement: Supplementary file 1 — Supplementary Information. [file 41598_2024_66884_MOESM1_ESM.docx]

Supplementary materials


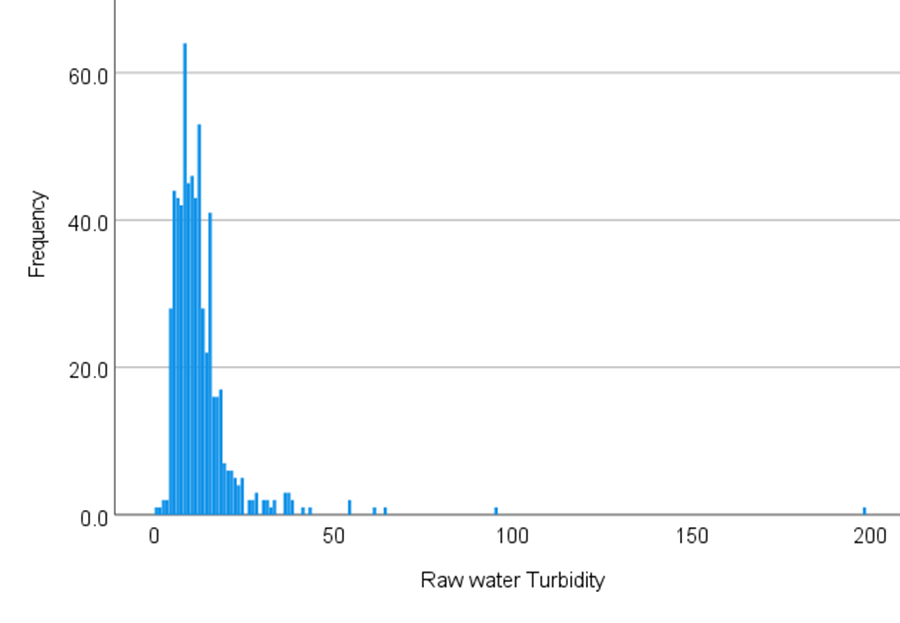


Figure SI-1 – Frequency distribution of raw water turbidity (NTU) during 2019-2020, regional Australia

Heavy rainfall

Bushfire event


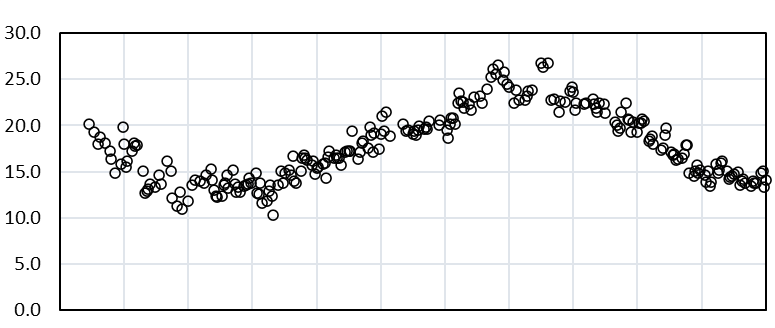


Temperature (C)


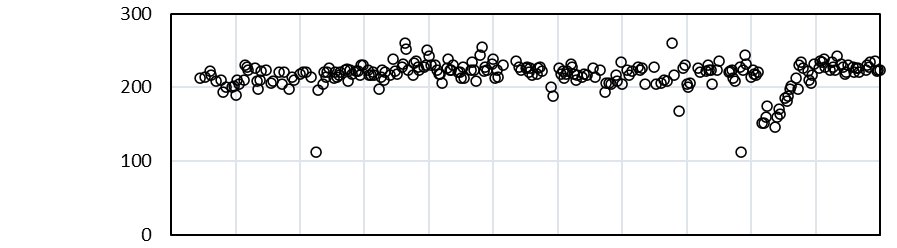


Total Hardness (mg/l)


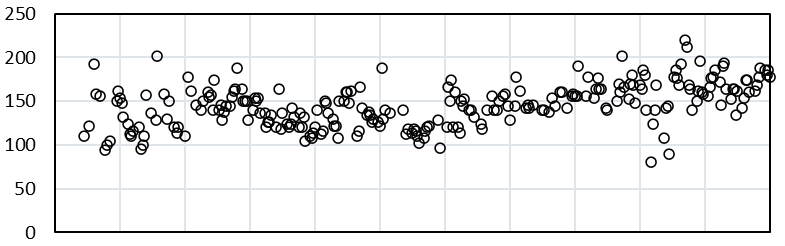


Alkalinity (mg/l)


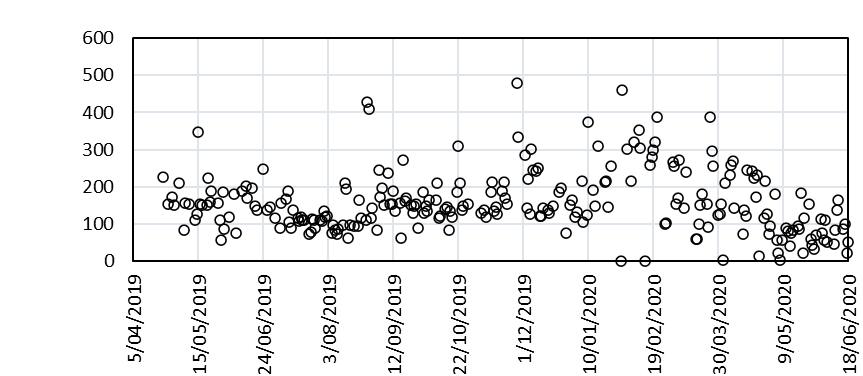


Colour (HU)

Figure SI-2 Variation in raw water temperature, total hardness, alkalinity and clour, and before and after the bushfire and rainfall event


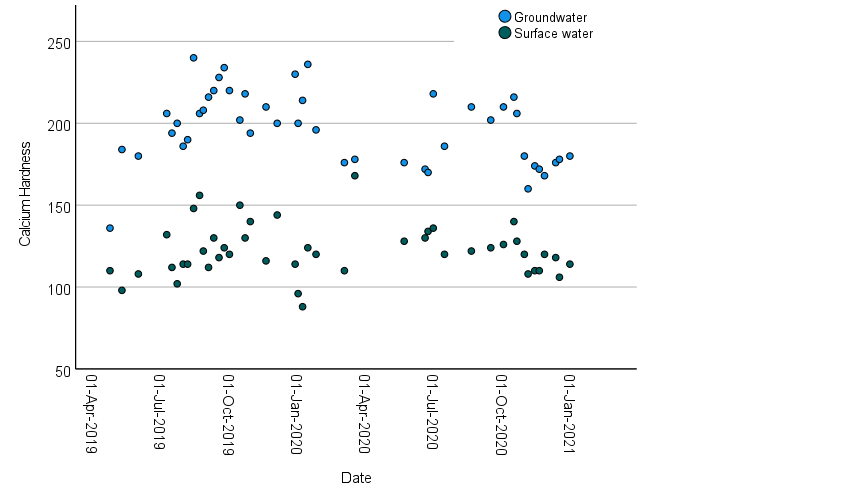


Figure SI-3- Variation of calcium hardness in groundwater and surface water

Table SI -4 (Treated Water Quality Targets & Guideline Values)

| Parameter | Units | ADWG | | Common Industry Treated Water Targets | Current Theodore Treated Water Target |
| --- | --- | --- | --- | --- | --- |
|  |  | Health | Aesthetic |  |  |
| Turbidity | NTU | 1 | 5 | < 0.1 | < 0.15 |
| Colour | HU |  | 15 | ≤ 5 | ≤ 5 |
| pH |  |  | 6.5 – 8.5 | 7.5 – 8.3 | 7.5-7.6 |
| Chlorine | mg/L | 5 |  | Depends on system | 1.0 – 1.5 |
| Total Aluminum | mg/L | 0.2 |  | ≤ 0.2 | - |
| Total Manganese | mg/L | 0.5 | 0.1 | ≤ 0.05 | - |
| Total Iron | mg/L |  | 0.3 | ≤ 0.3 | - |
| Total Alkalinity | mg/L as CaCO_3_ |  |  | ³ 40 | - |
| Total Dissolved Solids (TDS) | mg/L |  | < 600 | < 500 | - |
| Calcium Carbonate Precipitation Potential (CCPP) |  |  |  | -1 to -5 | - |
| Total Trihalomethanes | mg/L | 0.25 |  | 0.15 | - |

Figure SI-5: Free chlorine residual profiles in the treated water
